# Integrated analysis of genome-wide DNA methylation, gene expression and protein expression profiles in molecular subtypes of WHO II-IV gliomas

**DOI:** 10.1186/s13046-015-0249-z

**Published:** 2015-10-26

**Authors:** Zhi-Liang Wang, Chuan-Bao Zhang, Jin-Quan Cai, Qing-Bin Li, Zheng Wang, Tao Jiang

**Affiliations:** Department of Neurosurgery, Beijing Tiantan Hospital, Capital Medical University, No.6 TiantanXili, Dongcheng District, Beijing, 100050 China; Beijing Neurosurgical Institute, Capital Medical University, Beijing, China; Department of Neurosurgery, The Second Affiliated Hospital of Harbin Medical University, Harbin, China; Beijing Institute for Brain Disorders Brain Tumor Center, Beijing, China; China National Clinical Research Center for Neurological Diseases, Beijing, China

**Keywords:** Glioma, Subtype, Progression, Prognosis

## Abstract

**Background:**

Glioma is the most common malignant primary brain tumor among adults, among which glioblastoma (GBM) exhibits the highest malignancy. Despite current standard chemoradiation, glioma is still invariably fatal. A further insight into the molecular background of glioma is required to improve patient outcomes.

**Method:**

Previous studies evaluated molecular genetic differences through comparing different grades of glioma. Here, we integrated DNA methylation, RNA sequencing and protein expression data sets of WHO grade II to IV gliomas, to screen for dysregulated genes in subtypes during malignant progression of glioma.

**Results:**

We propose a list of universal genes (UG) as novel glioma biomarkers: 977 up-regulated genes and 114 down-regulated genes, who involved in cell cycle, Wnt receptor signaling pathway and fatty acid metabolic process. Poorer survival was associated significantly with the high expression of 977 up-regulated genes and low expression of 114 down-regulated in UG (*P* <0.001).

**Conclusion:**

To our knowledge, this was the first study that focused on subtypes to detect dysregulated genes that could contribute to malignant progression. Furthermore, the differentially expressed genes profile may lead to the identification of new therapeutic targets for glioma patients.

## Introduction

Glioma is the most common malignant primary brain tumor among adults [1, 2]. The tumors are graded on a WHO consensus-derived scale of I to IV according to their degree of malignancy, as judged by various histological features [3, 4]. Astrocytic tumors, which are the most common group of human gliomas, have an inherent tendency for recurrence and malignant progression, and usually cannot be cured by neurosurgical resection, radiotherapy and/or chemotherapy [5]. Regardless of treatment strategy, the majority of patients of low grade gliomas undergo recurrence or malignant transformation over time, and most patients eventually succumb to the disease. The 5-year progression rate of low grade gliomas was between 50 and 70 %, and malignancy-free survival rate of low grade gliomas was between 30 and 70 % [6, 7]. Relative survival estimates for glioblastoma are quite low. Only 5.0 % of patients survived five years after diagnosis [8]. Despite recent advances in cancer diagnosis and treatment, these statistics have not changed significantly. GBM still remains one of the most challenging cancers in clinical oncology [9].

Therefore, it is essential to investigate the mechanism involved in the development and progression of glioma. The Cancer Genome Atlas (TCGA) described a robust gene expression-based molecular classification of GBM into Proneural, Neural, Classical and Mesenchymal subtypes [10]. After that, TCGA indicated the existence of a glioma-CpG island methylator phenotype (G-CIMP). G-CIMP tumors, a subgroup of Proneural subtype, were more prevalent among lower-grade gliomas, displayed distinct copy-number alteration and were tightly associated with IDH1 somatic mutations [11]. IDH1 mutation, somatic mutation in isocitrate dehydrogenase 1 gene, occurs at high frequency in gliomas and seems to be a prognostic factor in glioblastoma patients [12–14]. Patients with G-CIMP tumors were younger at the time of diagnosis and had significantly improved outcome [15]. The GBM phenotype is ultimately a product of a set of processes, each prone to selective pressure and dysregulation in cancer. Selective pressure is accepted as a driving force behind cancer-associated remodelling of the genome, epigenome and proteome.

Previous studies evaluated molecular genetic differences through comparing different grades of glioma [16–18]. But the differences might be due to the different expression subtype, whose composition varied among grades. Here, we integrated DNA methylation, RNA sequencing and protein expression data sets of WHO grade II to IV glioma, to screen for dysregulated genes in subtypes during malignant progression. We found genes that could act universally in all subtypes of gliomas. Such an integrative approach is crucial for the identification of promising targets and the correponding therapies.

## Material & methods

### Patients and samples

High throughput data (DNA methylation, RNA sequencing and protein expression), molecular pathological data (IDH mutation) and clinical characteristics of astrocytic glioma patients (astrocytoma (A), anaplastic astrocytoma (AA) and glioblastoma multiforme (GBM)) were downloaded from The Cancer Genome Atlas (TCGA, http://cancergenome.nih.gov/). There were 195 samples in the methylation data; 340 samples in the RNAseq data and 149 samples in the protein expression data. The RNA sequencing data were log2 transformed before the following analysis.

### TCGA subtype annotation

TCGA subtypes were classified according to the Proneural-Neural-Classical-Mesenchymal classes using the signatures published in Verhaak et al. and single sample Gene Set Enrichment Analysis algorithm (ssGSEA) Glioma-CpG island methylator phenotype (G-CIMP) tumors were defined as IDH mutated proneural ones. The gliomas were classified into five different subtypes: G-CIMP-positive (IDH-mutated) proneural, G-CIMP-negative (IDH-wild-type) proneural, classical, mesenchymal and neural tumors.

### Differential analysis/Data mining

Significance analysis of microarrays (SAM) was applied to identify differentially methylated or expressed genes between two groups [19]. Because of the limited gene count in protein expression, the *t* test was used to determine differences in each two group comparison.

### Functional annotation

Gene Ontology (GO) analysis of differentially expressed genes was performed in DAVID (http://david.abcc.ncifcrf.gov/). Gene Set Enrichment Analysis (GSEA) was used to further validate the functional enrichment of those genes [20].

### Statistical analysis

Kaplan–Meier survival analysis was used to estimate the survival distributions, and the log-rank test was used to assess the statistical significance between stratified survival groups.

## Result

### Subtype annotation

**Fig. 1:**
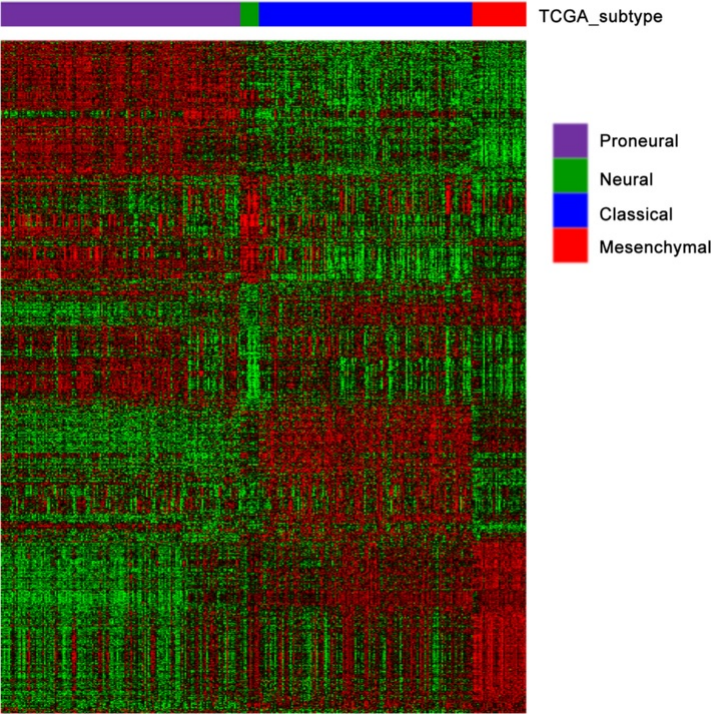
TCGA subtype annotation. Using the predictive 840 gene list, samples were ordered on the basis of subtype predictions

292 RNAseq data from TCGA samples (A, AA, GBM) were downloaded and were classified according to TCGA subtype signatures published by *Verhaak* et al. [21]. The proneural samples were futher classifed into G-CIMP-positive (IDH-mutated) proneural and G-CIMP-negative (IDH-wild-type) proneural accoding to IDH1/2 mutation status (Fig. 1). Because the limited number of neural and mesenchymal samples did not meet the threshold for statitical analysis, we focused on classical, IDH-mutated proneural and IDH-wild-type proneural subtypes in the following analysis. The sample size was listed in Table 1.

**Table 1:** Molecular pathology features of DNA methylation, RNAseq and protein expression samples

Database	DNA methylation			RNAseq			Protein expression		
Histology	A	AA	GBM	A	AA	GBM	A	AA	GBM
Total Number	20	35	112	48	96	151	22	47	58
Proneural IDH (+)	2	2	10	11	21	9	2	3	4
Proneural IDH (-)	13	12	16	23	35	16	13	24	10
Classical	3	18	86	8	36	89	4	18	44
Mesenchymal	0	3	0	1	4	30	0	1	0
Neural	2	0	0	3	2	7	3	1	0

### Genes universally contribute to malignant progression in three subtypes

We analyzed DNA methylation data and RNAseq data to find out differentially expressed genes that could universally contribute to malignant progression in three subtype. We used SAM to filter the high throughput data. Those genes were supposed to meet these following criteria: 1) differentially methylated and expressed between A and AA in three subtype (FDR < 0.05); 2) differentially methylated and expressed between A and GBM in three subtype (FDR < 0.05); 3) there was a negative correlation between its methylation and expression (Pearson’s correlation *p* value <0.05, *r* < 0).

The 1091 differentially expressed genes, containing 977 up-regulated and 114 down-regulated, were called universal genes (UG). Of the 172 genes with protein expression data, 9 genes (BRAF, RAF1, PDK1, BAK1, CCNE2, FN1, GATA3, IGFBP2, CCNB1) were differently expressed in protein level.

**Fig. 2:**
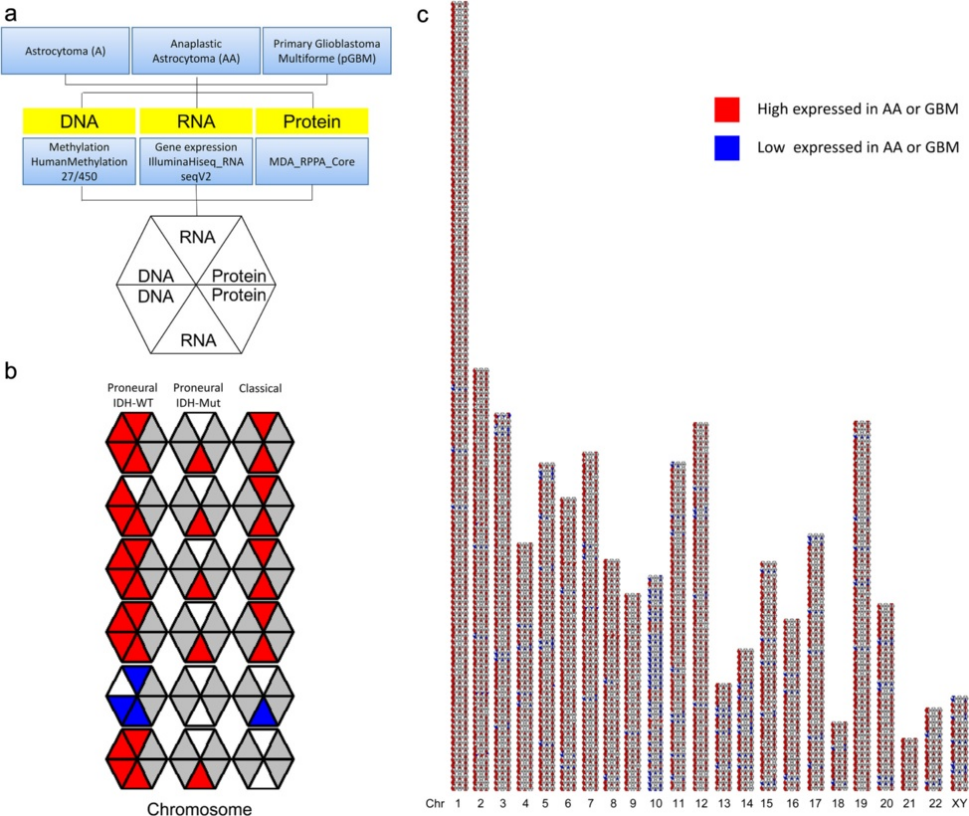
An integrated omic platform for the characterization of glioma patients in three subtypes. **a** A, AA and GBM were quantified for DNA methylation, RNAseq and protein expression. Data for each primary GBM and AA, relative to A, were color-encoded as indicated and integrated into six-sided polygons in a symmetrical manner, with AA data in the top three quadrants, and data from the GBM in the mirror-image bottom sections. **b** The polygons were assembled as a linear genetic map in the vertical direction, which organized by chromosome and by three subtypes in the horizontal direction: IDH-wild-type (IDH-WT) Proneural, IDH-mutated (IDH-Mut) Proneural, Classical. **c** By this approach 1091 data points were integrated into 3273 polygons. These reflected correlated changes in one or more of the linked genes across the set of individual AA and GBM. Red, high expression; Blue, low expression; White, not significant difference; Grey, data not available

The data were incorporated into a symmetrically divided hexagon, with color-encoded values for AA in the top quadrants and with corresponding values for the GBM in the bottom sections, as depicted in Fig. 2a. By this arrangement, the top and bottom halves of the hexagon represent mirror images of the DNA, RNA and protein measures for the AA and GBM, respectively.

In order to facilitate the recognition and visualization of genetically linked trends across tumours and grafts, an omic map was assembled in which 3273 hexagons (1091 genes × 3 subtypes) were assembled into an array by ordering horizontally according to 3 subtypes, and as a linear genetic map in the vertical direction (Fig. 2b and c).

**Fig. 3:**
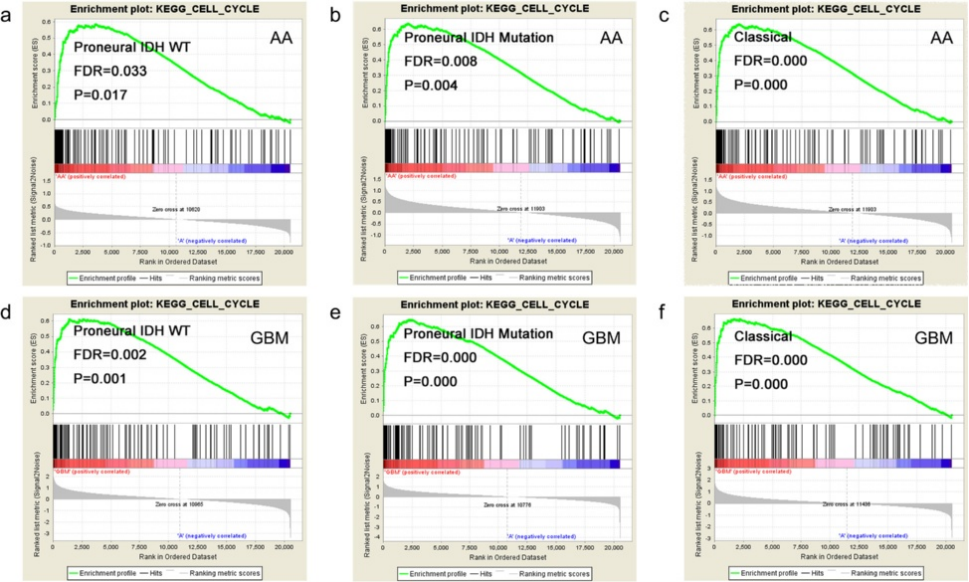
Gene set enrichment analysis of UG in three subtypes. The cell-cycle enrichment ploted of universally up-regulated genes in (**a**) IDH-wild-type Proneural AA, (**b**) IDH-mutated Proneural AA, (**c**) IDH Classical AA, (**d**) IDH-wild-type Proneural GBM (**e**) IDH-mutated Proneural GBM, (**f**) IDH Classical GBM

Gene Ontology analysis was performed by DAVID to infer the function of the differentially expressed genes. One hundred twenty-four up-regulated genes were enriched (*P* < 0.05 and FDR < 0.05) in cell cycle (Table 2). GSEA further validated the cell cycle association of the up-regulated genes in every subtype (Fig. 3). The down-regulated genes were enriched in Wnt receptor signaling pathway and fatty acid metabolic process.

**Table 2:** Top four GO terms of the 43 genes

GO term	Biological process	Gene count	*p-value*
GO:0007067	mitosis	34	1.44E-07
GO:0000280	nuclear division	34	1.44E-07
GO:0000087	M phase of mitotic cell cycle	34	2.21E-07
GO:0000279	M phase	43	3.11E-07

*GO* gene ontology

### Prognostic validation of universal genes in TCGA and external dataset

We examined prognostic value of UG on survival. The 1091 genes whose expression more strongly correlated with grade from TCGA database were used as markers to cluster 511 TCGA GBM samples for kmeans clustering (*k* = 2) [22, 23]. The prognosis of patients in group2 (highly expressed in up-regulated UG) (median: 386 days) was poorer than those in group1 (highly expressed in down-regulated UG) patients (median: 447 days, *p* = 0.0095) (Fig. 5a).

**Fig. 4:**
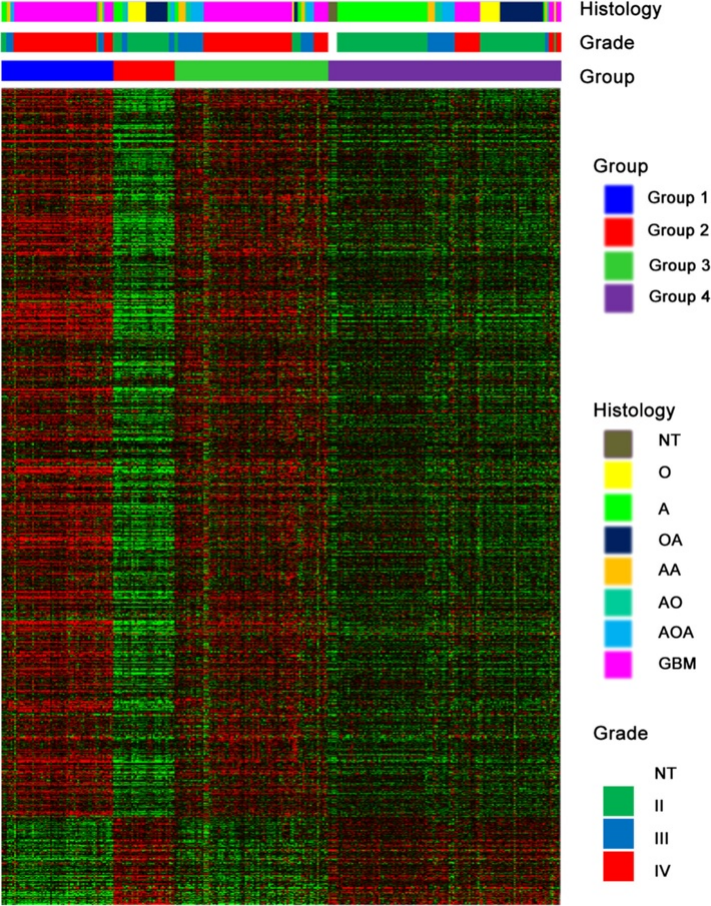
K-means clustering identified four groups of 310 CGGA samples by UG. K-means clustering identified of 310 CGGA samples by 1091 genes whose expression most strongly correlated with grade from TCGA database for *k* = 4

Next, we employed 310 glioma samples from the Chinese Glioma Genome Atlas (CGGA, http:// www.cgga.org.cn/) to validate the association between universal genes and overall survival using Kaplan–Meier survival curve (Fig. 4).

**Fig. 5:**
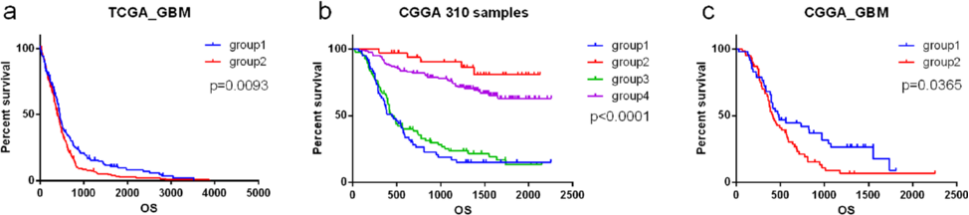
Kaplan-Meier estimated of survival for 511 TCGA and 310 CGGA samples. **a** Among 511 TCGA GBM samples, there was a significant difference in survival between two groups (*p* = 0.0095) (**b**) Among 310 CGGA samples, there was a significant difference in survival between four groups (*p* < 0.0001). **c** Among GBM patients in 310 CGGA samples, there was a significant difference in survival between two groups (*p* = 0.0365). Group1, high expression of down-regulated UG; Group2, high expression of up-regulated UG

The up-regulated UG were highly expressed in group 1 and 3, while down-regulated UG were highly expressed in group 2 and 4. There was a significant difference among the four groups. The OS was short in group1 (median: 447 days) and group 3 (median follow-up: 439 days). The median OS was un-reached in group 2 and group 4. Patients with high expression up-regulated UG indicated a significantly poor survival than those with high expression down-regulated UG (*p* < 0.0001) (Fig. 5b). Up-regulated UG expressed higher in Group1 than group3, and down-regulated UG expressed higher in group 2 than group 4 (Fig. 4). The results was concordant with the trend of survival curves, where the prognosis of these patients in group3 and group 2 were better than those in group1 and group4, respectively (Fig. 5b). However, the differences did not reach statistical significance. GBM (WHO grade IV) patients were mainly enriched in group1 and group2, while group2 and group4 showed highly association with oligodendroglioma (WHO grade II) and astrocytoma (WHO grade II).

To further confirm the result, we clustered the GBM samples in CGGA database by differentially expressed genes. The GBM samples were separated into 2 groups. High expression down-regulated UG also showed a better prognosis in patients (*p* = 0.0365) (Fig. 5c).

## Discussion

It is clear that GBM represents a heterogeneous type of neoplasm when considering its molecular and genetic features [24]. The integration of comprehensive data sets spanning the sequence to phenotype continuum DNA-RNA-protein-disease covered molecular signatures linking cancer progression and overall survival in glioma. Gliomas have been analyzed by several groups with the use of various types of large-scale methods. Low-grade astrocytomas (WHO grade 2), which account for approximately 35 % of human astrocytic tumors, generally affect young people with a mean age of 39 years. The most common chromosomal alteration seen in low-grade astrocytoma is the deletion of chromosome band 17p13.1 and mutations of the tumor suppressor gene p53 (TP53), which reside in this region [25]. Unlike low-grade astrocytomas, anaplastic astrocytomas are more cellular, have increased cellular atypia and have increased cellular proliferation [26]. The genetic changes associate with the transition from low-grade to anaplastic astrocytoma included allelic loss of chromosome arms 9p, 11p, 13q and 19q. Many of the alterations observe in anaplastic astrocytomas genes that regulate cell cycle progression. For example, alterations of the retinoblastoma (Rb) gene map to 13q14 occur in 40 % of anaplastic patients [18]. Glioblastomas are the most malignant astrocytic tumor. In addition to cellular atypia, increased mitotic index and infiltrative growth into adjacent normal brain, glioblastomas show intratumoral necrosis and vascular endothelial proliferation. Amplification of the EGFR locus is found in approximately 40 % of primary glioblastomas [27]. Mutations of the PTEN gene are found in 45 % of primary glioblastomas. Mutations of PTEN lead to constitutive activation of the phosphoti-dylinositol-3, 4, 5 triphosphate pathway; one member of this family is Akt. The mechanism of function and the role of PTEN in gliomagenesis have been widely studied [28].

These molecular genetic studied of gliomas detected molecular abnormalities through comparing different grades. However, we supposed that the molecular abnormalities distributed also associated significantly with subtypes. Here, we presented a particular study which focused on the same subtype in different grades to find out differentially expressed genes during malignant progression of glioma. Through this approach we discovered 1091 UG (977 up-regulated genes and 114 down-regulated genes) in DNA methylation and RNA sequencing data. The function of UG were mainly about cell cycle progression. Thus, that might be a good explanation for why patients with high expressed UG had a poor prognosis.

Because of the limitation of protein expression database, we discovered the UG mainly by analyze DNA methylation and RNA sequencing. After combining genes in protein expression database, only 9 up-regulated UG were left: BRAF, RAF1, PDK1, BAK1, CCNE2, FN1, GATA3, IGFBP2, CCNB1. There were six genes exhibited same tendency in A to AA and A to pGBM. The BRAF, RAF1, PDK1 had a lower protein expression in AA and GBM while BAK1, CCNE2, GATA3 had a higher protein expression. These data indicate that generally protein abundance may not be a simple relationship of DNA and mRNA levels, may reflect proteome remodelling in response to selective pressure for an aspect of the tumor phenotype that is manifest at the protein level.

We summarized 71 prognostic genes signature from previously papers [29–34]. The overlap of UG signature and previously genes signature were 19 genes. Most of the previously genes signature were based on high grade gliomas. In this paper, we integrated DNA methylation, RNA sequencing and protein expression data sets of WHO grade II to IV glioma, to screen for dysregulated genes, which were more accurate. There were 124 UG genes enriched (*P* < 0.05 and FDR < 0.05) in cell cycle. CCNB1, one of the UG genes, which is a protein essential for cell cycle progression through the G2/M phase. CCNB1 was reported as a novel therapeutic approach against many tumors [35–37] and may become novel therapeutic target for glioma.

## Conclusion

In summary, our study has provided a better understanding of tumor heterogeneity and disease progression in GBM. We show that up-regulated UG are a crucial factor for malignant progression and poor prognosis in glioma patients and modulate the cell cycle, and that down-regulation of UG predict a better outcomes in patients. To our knowledge, this is the first study that focus on subtypes to detect dysregulated genes that can contribute to malignant progression. Furthermore, the differentially expressed genes profile may lead to the identification of new therapeutic targets for glioma patients. Although the therapeutic opportunities for glioma remain limited, continuing efforts to detail the mechanisms of disease relapse would contribute to our ability to provide curative treatment for this lethal disease.
